# Increased epicardial tissue and reduced TAPSE and MAPSE scores in borderline personality disorders. Early indicators for cardiovascular risk?

**DOI:** 10.3389/fpsyt.2025.1441605

**Published:** 2025-06-03

**Authors:** Charlotte F. M. Schaefer, Britta Stapel, Nicole Scharn, Sebastian Bertele, Alexander Glahn, Kai G. Kahl, Phileas J. Proskynitopoulos, Mechthild Westhoff-Bleck

**Affiliations:** ^1^ Department of Psychiatry, Social Psychiatry and Psychotherapy, Hannover Medical School, Hannover, Germany; ^2^ Department of Cardiology and Angiology, Hannover Medical School, Hannover, Germany

**Keywords:** borderline personality disorder, epicardial adipose tissue, echocardiography, left ventricular function, right ventricular function, metabolic syndrome, lifestyle factors, cardiac function

## Abstract

**Introduction:**

Life expectancy of patients with borderline personality disorder (BPD) is reduced compared to the general population, which has been in part attributed to a heightened risk for cardiometabolic disorders. One prior study reported increased values of epicardial adipose tissue (EAT), which has been shown to be a sensitive marker for cardiovascular disease risk. Against this background, studies assessing cardiac function in patients with BPD have been missing to date.

**Methods:**

The present study included 28 female patients with a diagnosis of BPD and 28 age, sex, and BMI-matched controls (mean age 29 ± 11 years). EAT thickness and parameters of cardiac function were assessed by echocardiography. Diabetes risk was assessed using the Finnish Diabetes Risk (FINDRISC) score, and metabolic syndrome was defined in accordance to National Cholesterol Education Program Adult Treatment Panel-III (NCEP/ATPIII) criteria. Additionally, self-report questionnaires were used to assess lifestyle factors, retrospectively reported childhood trauma and current symptoms of depression and anxiety.

**Results:**

Our study confirmed significantly elevated levels of EAT in patients with BPD compared to controls. Additionally, significant decreases in right (TAPSE) and left (MAPSE) ventricular function, albeit within the normal range, were measured in BPD patients. Contrarily, left ventricular ejection fraction was similar in both groups. Further, patients with BPD reported high levels of childhood trauma and clinically relevant depression and anxiety symptoms. Diabetes risk and frequency of metabolic syndrome as well as serum levels of prognostic markers NT-proBNP and GDF15 were similar in both groups. BPD patients were more frequently smokers and reported lower levels of physical exercise compared to controls.

**Conclusion:**

The present study demonstrates morphological and functional differences in a matched sample of female patients with BPD and healthy controls, pointing to an increased risk for the development of cardiovascular disorders. These findings highlight the importance of screening for cardiovascular risk markers and of including interventions that aim to improve adverse life-style habits early on in the clinical management of BPD.

## 1 Introduction

Borderline personality disorder (BPD) is characterized by a distorted self-image, instability in mood, impaired emotion regulation, and chronic feelings of inner emptiness (1). Patients with BPD are often unable to develop and maintain healthy relationships, frequently divorce, display chronic behavior of self-harm, substance abuse and dependence, and are frequently hospitalized, have difficulties maintaining a job, have a reduces self-care and are often chronically suicidal. Taken together, this accumulates in a negative perception of individuals with BPD by health practitioners as well as by the general public. In this regard, the stigma associated with the diagnosis of BPD appears to go beyond that associated with other mental disorders (2) and consequently individuals with BPD often report continued struggles in seeking help, receiving adequate and consistent care, and being understood (3).

Borderline personality disorder has an international lifetime prevalence of approximately 2% worldwide (4). Patients with BPD have a reduced life expectancy of approximately 10–20 years (5, 6). While BPD is associated with high suicide rates compared to other psychiatric disorders, i.e. between 46% and 92% of BPD patients attempt suicide at least once in their lifetime (7), with 3-10% dying by suicide (8), it is important to note that suicide explains only a small part of the observed reduction in life expectancy. A dedicated study found that over a 24-year follow-up, 14% of individuals with BPD died from causes other than suicide, while 5.9% of deaths could be attributed to suicide (8).

Among the cited reasons for the substantial reduction in life expectancy are a sedentary lifestyle, obesity, atherosclerosis, diabetes, hypertension, and metabolic syndrome (MetS), which commonly constitute risk factors for cardiovascular disease (CVD) (9–12). Accordingly, a significantly elevated risk for coronary heart disease and stroke respectively have been described in the context of BPD when compared to the general population (13).

Scientific studies have shown that intra-abdominal adipose tissue (IAT) constitutes an independent risk factor for cardiovascular and metabolic morbidity and mortality (14). Epicardial adipose tissue (EAT) is a specialized type of adipose tissue that surrounds the heart, located between the epicardium and the pericardium (15). It has gained attention in CVD research due to its potential involvement in the pathophysiology of various CVDs (i.e. coronary artery disease, atrial fibrillation, cardiac arrhythmia, and heart failure) (16). In addition, abnormal metabolic activity or an increased volume of EAT could be associated with a higher risk of cardiovascular events and pathological changes, including hypertrophy, failure to store triglycerides and elevated lipolysis and inflammation (17). Epicardial adipose tissue has previously been found to be increased in BPD compared to healthy individuals but also in comparison to patients with major depression (18). In line with these findings, an elevated CVD risk has been reported that appears to be specific for BPD in comparison to other personality disorders even when controlling for comorbid depression (13, 19).

However, as EAT constitutes only a structural parameter associated with CVD, one could ask if functional parameters, such as ejection fraction (EF) and additional measurements of left and right ventricular function, may be reduced in the context of BPD. Studies regarding cardiac function in patients with BPD are scarce. In this regard, a recent study reported greater global longitudinal stress, which constitutes an echocardiographic marker for left ventricular deformation and a suitable indicator for CVD, in younger (age 20–40 years) female patients with BPD compared to healthy controls (20). We are unaware of studies that compared EAT as well as parameters of cardiac function in healthy controls and BPD patients.

The aims of the present study were as follows: We applied echocardiography, to replicate and expand on our previous study that reported increased EAT values in patients with BPD (18). Further we aimed to expand on our previous findings by assessing parameters of cardiac function and factors of metabolic syndrome (MetS) in individuals with BPD compared to healthy controls, matched by sex, age and BMI. We hypothesized that individuals diagnosed with BPD may display increased EAT thickness. Furthermore, we hypothesized that parameters of left and right ventricular function might be decreased in individuals with BPD compared to healthy controls.

## 2 Materials and methods

### 2.1 Subjects

The Hannover Medical School Ethics Committee reviewed and approved the present prospective case-control study (Ethic number: 5750). All participants provided written informed consent, and the procedures followed the principles of the Declaration of Helsinki. Patients with BPD were currently or have been previously treated at the Department of Psychiatry, Social Psychiatry, and Psychotherapy of Hannover Medical School. Inclusion criterion for the BPD group was a diagnosis of BPD made by a psychiatrist and/or psychotherapist following the Diagnostic and Statistical Manual of Mental Disorders guidelines, fifth edition (DSM-5) (21). Accordingly, all patients in the BPD group met the diagnostic criteria for BPD according to DSM-5, and a corresponding diagnosis (ICD-10 code F60.31) was documented in each patient’s medical record. The participants in the control group did not exhibit any apparent mental illness at the time of the study based on individual anamneses. Exclusion criteria for the study were male gender, pregnancy, taking antibiotics or proton pump inhibitors within the past two weeks, immune or autoimmune disease, lifelong or current cardiovascular disease, mental disability, schizophrenia, bipolar disorder, current abuse of any substances, and age under 18 or over 60 years. N=28 individuals, matched for age and BMI, were included in the BPD group and the control group, respectively.

### 2.2 Behavioral assessment

Depression and anxiety were determined by the use of the German version of the Hospital Anxiety and Depression Scale (HADS). The severity of anxious and depressive symptoms during the past two weeks was assessed by self-report by use of two subscales with seven items each (22, 23). Lifestyle factors were collected through self-report. Participants were asked about their weekly alcohol consumption, daily cigarette use, and frequency of physical activity on a scale of 1 (no activity) to 6 (very often, more than three times a week) (24).

The study employed the Childhood Trauma Questionnaire (CTQ) to evaluate the physical and psychological abuse experienced by the participants during their childhood. Participants rated the frequency of maltreatment on a five-point scale ranging from 1 (not at all) to 5 (very frequently). The questionnaire comprises five subscales: emotional abuse, physical abuse, emotional neglect, physical neglect, and sexual abuse (25).

### 2.3 Blood sampling, metabolic syndrome and diabetes risk score

Abdominal circumference, height, weight, and blood pressure were collected. The Finnish Diabetes Risk (FINDRISC) score was utilized to identify patients with an elevated risk of developing type II diabetes mellitus within the next decade (26). Serum samples were collected at the same day as the cardiac assessment took place. Samples were aliquoted and stored at -80°C for further processing. The following metabolic markers were measured in serum samples by use of routine laboratory methods: triglycerides, high-density lipoprotein (HDL) cholesterol, low-density lipoprotein (LDL), cholesterol, and glucose. Further, serum levels of the diagnostic and prognostic cardiac markers growth/differentiation factor 15 (GDF15) and N-terminal prohormone of brain natriuretic peptide (NT-proBNP) were determined. Presence of metabolic syndrome was assessed following the National Cholesterol Education Adult Treatment Panel-III R (NCEP/ATP III) criteria (27).

### 2.4 Cardiac assessment

All participants underwent a comprehensive examination by a specialized cardiologist that included echocardiography. The echocardiographic assessment of EAT was performed using two-dimensional standard parasternal long-axis/short-axis views at the end of the diastole. EAT thickness was measured on the right ventricular free wall perpendicular to the aortic annulus (28). To investigate differences in cardiac function, the following parameters were determined using transthoracic echocardiography: Tricuspid annular plane systolic excursion (TAPSE) was used to measure right ventricular function. It is assessed by TTE with M-Mode from the apical window (29). Mitral annular plane systolic excursion (MAPSE) is a simple method for evaluating left ventricle function (30). The ejection fraction (EF) is a measure of the percentage of the total volume that is pumped from the left ventricle into the systemic circulation during systole.

### 2.5 Statistical analysis

Statistical analyses were performed using SPSS 28 (IBM, Armonk, NY, USA). Sample size calculation was carried out by use of G*Power 3.1 (31, 32) as specified in **Supplementary Table S1**. Normality of continuous data was tested by use of the Shapiro-Wilk test. Group comparisons of categorical data were carried out by use of the Chi-Square test, and comparison of continuous data (i.e., anthropometric data, lifestyle factors, cardiometabolic risk and serum factors) was performed by Mann-Whitney U test. Univariate analysis of covariance (ANCOVA), with the inclusion of age and BMI as covariates, was performed to assess group differences regarding EAT- as well as parameters of cardiac function. Two-tailed p-values are depicted, and p≤.05 was considered statistically significant.

## 3 Results

### 3.1 Demographic parameters and lifestyle factors

The combined sample’s characteristics regarding age, body composition, and cardiovascular parameters, including BPD patients and CTRLs, are summarized in **Supplementary Table S2**. BPD patients in the present sample frequently indicated symptoms of depression and anxiety as well as high levels of retrospectively reported childhood trauma (**Supplementary Table S3**).

The following results regarding demographic, anthropomorphic and cardiometabolic data and lifestyle behaviors in the BPD and CTRL group are depicted in **Table 1**. As patients and CTRLs were matched for age and BMI, no significant differences were observed between both groups with regard to these parameters (age: *U*=340.0, *Z*=-.856, *p*=.392; BMI: *U*=368.5, *Z*=-.385, *p*=.700). Compared to CTRLs, BPD patients smoked more frequently (χ²(1)=23.468, φ=.659, *p*<.001) and reported less physically exercise (*U*=106.0, *Z*=-4.206, *p*<.001). No significant differences were observed concerning alcohol consumption (*U*=293.5, *Z*=-.302, *p*=.763) and no significant difference in diabetes risk, assessed by FINDRISC, was observed between groups, with the majority of participants displaying scores indicating a risk of 4% or below (BPD: 88.9% vs. CTRL: 88.0%). This was reflected by similar median values within the low-risk area (*U*=260.0, Z=-1.433, *p*=.152). Similarly, no statistically significant group difference was found in serum levels of the prognostic markers NT-proBNP (*U*=289.5, *Z*=-1.644, *p*=.100) and GDF15 (*U*=372.0, *Z*=-.101, *p*=.919) with mean levels found to be within the normal range in both groups. Finally frequency of metabolic syndrome (χ²(1)=.000, φ=.000, *p*=1.000) did not significantly differ between groups. Further, none of the criteria for metabolic syndrome differed significantly (**Supplementary Table S4**).

**Table 1:** Comparison of demographic, anthropomorphic and cardiometabolic data and lifestyle behaviors in borderline personality disorder patients and controls.

Parameter	CTRL	BPD	Statistics
Age (years)	25.0 [22-33.8]	23.5 [21.0-30.8]	*U*=340.0, *Z*=-.856, *p*=.392 (a)
BMI (kg/m^2^)	23.8 [21.7-26.0]	23.5 [20.6-26.8]	*U*=368.5, *Z*=-.385, *p*=.700 (a)
Smoking (*N* [%])	1 [3.7%]	18 [66.7%]	χ²(1)=23.468, φ=.659, *p*<.001 (b)
Alcohol consumption (drinks/week)	1 [0-3]	0.5 [0-2]	*U*=293.5, *Z*=-.302, *p*=.763 (a)
Physical exercise	5 [4-6]	3 [2-4]	*U*=106.0, *Z*=-4.206, *p*<.001 (a)
Diabetes Risk (FINDRISC)	3 [0.5-7.5]	4 [3.0-11.0]	*U*=260.0, Z=-1.433, *p=*.152 (a)
MetS (*N* [%])	4 [14.8%]	4 [14.8%]	χ²(1)=.000, φ=.000, *p*=1.000 (b)
NT-proBNP (ng/l)	51 [49-88]	49 [49-68]	*U*=289.5, *Z*=-1.644, *p*=.100 (a)
GDF15 (ng/l)	619 [446-828]	606 [413-794]	*U*=372.0, *Z*=-.101, *p*=.919 (a)

Continuous data are depicted as median with interquartile range. Categorical data are shown as N-number and percentages of total. BMI, body mass index; FINDRISC, Finnish Diabetes Risk Score; GDF15, growth/differentiation factor 15; MetS, Metabolic Syndrome; NT-proBNP, N-terminal prohormone of brain natriuretic peptide.

(a) Mann-Whitney U Test.

(b) Chi-Square Test.

### 3.2 Epicardial adipose tissue thickness and parameters of cardiac function

**Figure 1:**
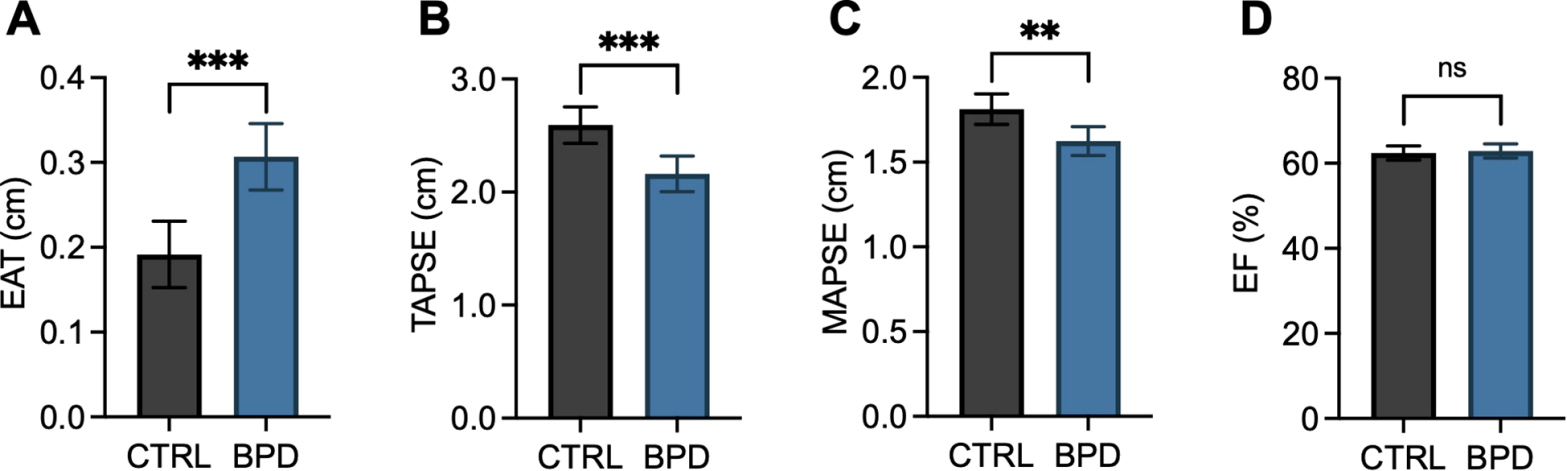
Epicardial adipose tissue thickness and cardiac function parameters in patients with borderline personality disorder compared to controls. Bar graphs depict estimated marginal means and 95% confidence intervals calculated by univariate ANCOVAs with age and BMI as covariates. Epicardial adipose tissue thickness (EAT, **A**), right ventricular systolic function (TAPSE, **B**), left ventricular systolic function (MAPSE, **C**), and left ventricular ejection fraction (EF, **D**) are depicted. Two-tailed p-values are shown, and p≤.05 was considered statistically significant. ***p<.001; **p≤.01; ns, not significant.

Potential group effects on prognostic and functional cardiovascular markers were tested by use of univariate ANCOVAs with age and BMI as covariates. Epicardial adipose tissue thickness was significantly increased in patients with BPD compared to CTRLs (*F*(1, 52)=17.398, *p*<.001; **Figure 1A**). Regarding functional parameters, while within the normal range in both groups, right ventricular systolic function (TAPSE: *F*(1, 51)=14.735, *p*<.001; **Figure 1B**) and left ventricular systolic function (MAPSE: *F*(1, 49)=9.240, *p*=.004; **Figure 1C**) were significantly decreased in BPD patients compared to CTRL. Conversely, no significant difference was found regarding left ventricular ejection fraction (EF: *F*(1, 52)=4.771, *p*=.621; **Figure 1D**).

Importantly, the group effect on EAT remained significant after adding measures of alcohol consumption and systolic blood pressure as covariates to the ANCOVA (F(1, 43)=15.674, *p*<.001).

## 4 Discussion

In the present study, we found morphological and functional between-group differences concerning EAT and cardiac function, demonstrating that patients with BPD are at increased risk for developing cardiovascular disorders. In particular, EAT thickness was increased and cardiac function was decreased in a sex, age, and BMI-matched sample of patients with BPD compared to healthy controls. Regarding MAPSE and TAPSE scores, functional differences were found, with no differences in ejection fraction. Physical activity was lower and smoking frequency higher in BPD, while there was no significant difference in the frequency of metabolic syndrome, diabetes risk, and alcohol consumption.

Here, we replicated the findings of our previous study in which we reported increased EAT volume in BPD and discussed whether EAT could be an early predictor and potential mediator of CVD in BPD (18). Several studies have identified and confirmed an increased CVD risk of BPD patients (13, 19). This might be partly attributable to underlying factors, such as adverse childhood experiences, that have been proposed to be associated with emotional dysregulation and impulsivity in the BPD (33), but were also found to be associated with ischemic heart disease (34). Of note, one community study has found BPD to be independently associated with CVD risk indicators, concluding that future studies and therapies should aim to identify interventions to affect behavioral and biological mechanisms in BPD (19).

In contrast to our previous study (18) in which participants were matched for age and sex, in the present study patients and controls were additionally matched for BMI as these factors have been previously reported to be associated with EAT content (35–37). Despite this stringent matching, we found EAT thickness to be significantly increased in the BPD group, while there were no differences in metabolic syndrome, diabetes risk, or alcohol consumption. Regarding other lifestyle factors that have a potential influence on cardiovascular risk, we found increased smoking frequency and lower physical activity in the BPD group. Interestingly, those differences did not directly translate into significantly higher risks for diabetes or higher frequency of metabolic syndrome but, arguably, into higher EAT thickness.

A recent study by Gustafsson and colleagues that assessed determinants of EAT in a general middle-aged population (N=1,667; age 33–49 years), reported a direct association of waist circumference, systolic blood pressure, and red meat intake with EAT thickness in the complete sample of male and female participants (38). Contrarily, age and alcohol consumption were correlated with EAT only in women (38). Data regarding dietary habits were not queried in the present study and smoking status was only queried as smoking or non-smoking and was therefore not included in the ANCOVA. However, in the present sample that included only female participants, controlling for age, BMI, systolic blood pressure and weekly alcohol consumption did not diminish the robust association of EAT thickness and BPD.

Thus, having in mind a generally increased risk for CVD in BPD, it appears reasonable to argue that EAT content could indeed serve as an early marker for mid-life and late-life cardiovascular risk.

MAPSE is a relatively sensitive marker for left ventricular function (39, 40) and TAPSE, respectively, for right ventricular function, with the latter having been shown to predict cardiac death in the general population (41). In our sample, we identified significant differences in MAPSE and TAPSE values, while there were no differences in ejection fraction. This could be explained by the fact that, even though we found a difference in both scores, average values were still below the pathological threshold usually applied (normal range MAPSE: > 10 mm; normal range TAPSE: > 17 mm (42)). Nonetheless, against the background that BPD is associated with increased cardiovascular risk, our results show early but not yet pathological changes in cardiac function in BPD when compared to healthy controls. Considering that as pointed out above, reduced TAPSE values were found to be associated with earlier cardiac death in the general population, our findings underline the observed association of BPD with CVD (13).

It is evident throughout the general and scientific community that exercise is a fundamental tool for reducing cardiovascular risk (43, 44), and smoking is one of the critical risk factors for CVD (45). Furthermore, physical exercise strongly affects cardiac function and echocardiographic measurements (43, 44, 46). Thus, the differences found in our study could be at least partly linked to the observed differences in lifestyle.

However, arguably those differences are in some capacity inherent to BPD. According to Bohus (2011), the psychopathology of BPD unfolds in three dimensions comprising all diagnostic criteria found in the DSM-5, namely disturbances in the regulation of the affective state, disturbances in identity and disturbances in social interactions (21, 47). Impulsivity is directly linked to measures of self-harm often found in BPD, including substance abuse and binge eating. Arguably, emotional instability in reaction to day-to-day events, chronic feelings of emptiness, and impulsivity in general are three factors that compromise upholding a healthy and more functional lifestyle in the long run. Investigations of learning processes have shown disturbed emotional learning in BPD (48), especially during dissociative states (49). From a psychotherapeutic perspective, this is a critical factor when it comes to starting and upholding functional behavior such as physical exercise or smoking cessation. Disturbed learning processes can attenuate the positive effect of behavioral activation on mood and well-being, thereby decreasing the probability of upholding this behavior.

Regarding its etiology, BPD is understood to develop as a result of the interaction of genetic factors and adverse childhood events (4, 50). Our research group recently outlined the role of adverse childhood events on cardiovascular health. In a study on 210 congenital heart disease outpatients and using mediation analysis, we have shown that adverse childhood events are linked to increased depressive symptoms, which are related to decreased physical activity, which in turn are connected to a higher EAT content (51). Depressive symptoms are frequently linked to BPD and often as high as severe major depression while usually being phenomenologically different (52). Over 50% of BPD patients in our sample presented with pathologic depression and anxiety symptoms based on self-report. Further, levels of childhood trauma were high, with over 50% of patients retrospectively reporting at least moderate to severe levels of neglect and/or abuse in each of the five domains of the CTQ. Thus, our results appear in line with the previously reported findings of the above described mediation analysis, indicating that BPD is associated with lower physical exercise and more tobacco use which might contribute to increased EAT thickness and additionally to mild decreases in cardiac function.

### 4.1 Limitations

This study has several limitations. Although our samples were matched for sex, age and BMI, the differences could partly be explained by physical activity and tobacco use, even though we can assume the latter is a consequence of BPD. Furthermore, external validity is reduced by the relatively small sample size. All patients were recruited from the outpatient clinic or the psychotherapeutic ward of a university hospital in Germany. Thus, the functional level can be regarded as relatively high compared with those patients who are usually unable to attend dialectic-behavioral therapy due to frequent suicide attempts or severe and life-threatening self-harm. As there was no documentation regarding the history of psychopharmacological treatment available, it cannot be excluded that long-term use of psychiatric medication could have an impact on the assessed outcome parameters. Furthermore, since we only included females in our analysis, we cannot extrapolate our findings to persons of the male sex, although it is fair to assume that we would see the same effects.

## 5 Conclusion

In summary, we found increased EAT and reduced MAPSE and TAPSE values in a sample of age, sex, and BMI-matched persons with BPD compared with healthy controls. This study highlights the relationship between BPD as a risk factor for a particular lifestyle, which is associated with increased cardiovascular risk. Furthermore, we present evidence that EAT could be an objective prognostic marker for CVD in BPD before other factors, such as diabetes risk or metabolic abnormalities, are significantly different.

## Data Availability

The raw data supporting the conclusions of this article will be made available by the authors, without undue reservation.
