# Supplementary material for: Increased epicardial tissue and reduced TAPSE and MAPSE scores in borderline personality disorders. Early indicators for cardiovascular risk?
Source: Front Psychiatry. 2025 Jun 3;16:1441605. doi: 10.3389/fpsyt.2025.1441605 (PMC12175066; doi:10.3389/fpsyt.2025.1441605)
Supplement: Supplementary file 1 [file DataSheet1.pdf]

Supplementary Material

1 Supplementary Tables

Supplementary Table S1: Input parameters for sample size calculation using G*Power 3.1

Test family	F test
Statistical test:	Fixed effects, main effects and interactions
Type of power analysis	A priori: Compute required sample size – given α, power, and effect size
Effect size f	0.4
α error probability	0.05
Power (1-β error probability)	0.8
Number of groups	2
Number of covariates	2

Supplementary Table S2: Demographic and anthropomorphic data, lifestyle factors, and cardiovascular parameters of the study sample

Age (years)	28.8 ± 11.1	24 [22-31]
BMI (kg/m^2)	24.2 ± 4.3	23.7 [21.3-26.6]
Smoking (*N* [%])	19 [35.2%]	
Alcohol consumption (drinks/ week)	1.5 ± 2.1	1.0 [0-2.3]
Physical exercise	3.68 ± 1.56	3.5 [2.0-5.0]
Diabetes Risk (FINDRISC)	5.63 ± 5.32	4.0 [3.0-9.5]
MetS (*N* [%])	8 [14.8%]	
NT-proBNP (ng/l)	69.3 ± 35.5	49.0 [49.0-75.0]
GDF15 (ng/l)	653.3 ± 250.4	618.0 [448.0-790.0]
TAPSE (cm)	2.37 ± 0.47	2.30 [2.00-2.65]
MAPSE (cm)	1.71 ± 0.25	1.70 [1.50-1.90]
EF (%)	62.6 ± 4.4	62.0 [60.0-65.5]
EAT (cm)	0.25 ± 0.14	0.21 [0.15-0.30]

Continuous data are depicted as means ± standard derivation and as median with interquartile range. Categorical data are shown as N-number and percentage of total. BMI, body-mass index; EAT, epicardial adipose tissue; EF, ejection fraction; FINDRISC, Finnish Diabetes Risk Score; GDF15, growth/differentiation factor 15; MAPSE, mitral annular plane systolic excursion; MetS, Metabolic Syndrome; NT-proBNP, N-terminal prohormone of brain natriuretic peptide; TAPSE, tricuspid annular plane systolic excursion.

Supplementary Table S3: Psychometric parameters and levels of retrospectively reported childhood trauma in patients with borderline personality disorder.

HADS	
Depression (*N* [%])	normal: 7 [25%]<br>elevated: 5 [18%]<br>pathologic: 15 [54%]
Anxiety (*N* [%])	normal: 2 [7%]<br>elevated: 6 [21%]<br>pathologic: 18 [64%]
CTQ	
Emotional abuse (*N* [%])	none to minimal: 1 [4%]<br>low to moderate: 6 [21%]<br>moderate to severe: 8 [29%]<br>severe to extreme: 13 [46%]
Physical abuse (*N* [%])	none to minimal: 15 [54%]<br>low to moderate: 3 [11%]<br>moderate to severe: 3 [11%]<br>severe to extreme: 7 [25%]
Sexual abuse (*N* [%])	none to minimal: 9 [35%]<br>low to moderate: 4 [14%]<br>moderate to severe: 8 [29%]<br>severe to extreme: 5 [18%]
Emotional neglect (*N* [%])	none to minimal: 5 [18%]<br>low to moderate: 5 [18%]<br>moderate to severe: 4 [14%]<br>severe to extreme: 14 [50%]
Physical neglect (*N* [%])	none to minimal: 8 [29%]<br>low to moderate: 6 [21%]<br>moderate to severe: 4 [14%]<br>severe to extreme: 10 [36%]

N-numbers and percentage of total are depicted. Severity levels were determined in accordance to dedicated literature. CTQ, childhood trauma questionnaire; HADS, hospital anxiety and depression scale.

Supplementary Table S4: Comparison of metabolic syndrome criteria in accordance to NECP ATP III in controls and patients with borderline personality disorder.

	CTRL	BPD	Statistics
BP_{sys} (mmHg)	129 ± 13	127 ± 12	$t(53) = .606$, $p = .547$ (a)
BP_{dia} (mmHg)	81 ± 9	80 ± 10	$t(53) = .269$, $p = .789$ (a)
Waist circumference (cm)	80 ± 11	81 ± 12	U=365.5 Z=-.211, p=.833 (b)
Triglycerides (mmol/l)	1.36 ± 0.63	1.33 ± 0.88	U=323.0, Z=-.926, p=.354 (b)
HDL (mmol/l)	1.74 ± 0.42	1.53 ± 0.37	$t(53) = 1.951$, $p = .056$ (a)
Glucose (mmol/l)	4.7 ± 0.67	4.8 ± 1.00	U=347.0, Z=-1.482, $p = .601$ (a)

Means ± standard deviations are depicted. BPsys, systolic blood pressure; BPdia, diastolic blood pressure.

(a) *t* test

(b) Mann-Whitney-U Test
